# DM1 Transgenic Mice Exhibit Abnormal Neurotransmitter Homeostasis and Synaptic Plasticity in Association with RNA Foci and Mis-Splicing in the Hippocampus

**DOI:** 10.3390/ijms23020592

**Published:** 2022-01-06

**Authors:** Brigitte Potier, Louison Lallemant, Sandrine Parrot, Aline Huguet-Lachon, Geneviève Gourdon, Patrick Dutar, Mário Gomes-Pereira

**Affiliations:** 1 LuMIn, CNRS FRE2036, ENS Paris-Saclay, CentraleSupelec, Université Paris-Saclay, 91190 Gif-sur-Yvette, France; brigitte.potier@universite-paris-saclay.fr (B.P.); patrick.dutar@universite-paris-saclay.fr (P.D.); 2 Centre de Recherche en Myologie, Institut de Myologie, Inserm, Sorbonne Université, 75013 Paris, France; louison.lallemant@inserm.fr (L.L.); aline.huguet@inserm.fr (A.H.-L.); 3 Lyon Neuroscience Research Center, Inserm U1028, CNRS UMR5292, Université Lyon 1, 69500 Bron, France; sandrine.parrot@univ-lyon1.fr

**Keywords:** myotonic dystrophy, brain pathology, synaptic plasticity, neurotransmission, glutamate, GABA, neurotransmitter uptake, RNA splicing, transgenic mouse model

## Abstract

Myotonic dystrophy type 1 (DM1) is a severe neuromuscular disease mediated by a toxic gain of function of mutant RNAs. The neuropsychological manifestations affect multiple domains of cognition and behavior, but their etiology remains elusive. Transgenic DMSXL mice carry the DM1 mutation, show behavioral abnormalities, and express low levels of GLT1, a critical regulator of glutamate concentration in the synaptic cleft. However, the impact of glutamate homeostasis on neurotransmission in DM1 remains unknown. We confirmed reduced glutamate uptake in the DMSXL hippocampus. Patch clamp recordings in hippocampal slices revealed increased amplitude of tonic glutamate currents in DMSXL CA1 pyramidal neurons and DG granule cells, likely mediated by higher levels of ambient glutamate. Unexpectedly, extracellular GABA levels and tonic current were also elevated in DMSXL mice. Finally, we found evidence of synaptic dysfunction in DMSXL mice, suggestive of abnormal short-term plasticity, illustrated by an altered LTP time course in DG and in CA1. Synaptic dysfunction was accompanied by RNA foci accumulation in localized areas of the hippocampus and by the mis-splicing of candidate genes with relevant functions in neurotransmission. Molecular and functional changes triggered by toxic RNA may induce synaptic abnormalities in restricted brain areas that favor neuronal dysfunction.

## 1. Introduction

Myotonic dystrophy type 1 (DM1) is a multisystemic disease that affects patients of all ages [1,2]. In addition to typical muscle symptoms, the central nervous system (CNS) is critically impaired. The debilitating neurological manifestations range from marked intellectual disability in the congenital form; low processing speed, attention deficits, and cognitive impairment in childhood patients; to typical executive dysfunction in adults [3,4], which may be associated with increased anxiety, depression, and/or anhedonia [5]. Cognitive and behavioral impairment is accompanied by a broad spectrum of imaging changes widely dispersed throughout the brain [6]. Brain structural abnormalities include prevalent white matter lesions [7], some of which may denote focal demyelination [8]. Reduced grey matter volume has also been reported in cortical brain regions and in the hippocampus of DM1 patients [7,9,10], corroborating histopathological evidence of diffusing cerebral atrophy and neuronal loss [11,12]. It is conceivable that brain structural abnormalities in DM1 result in alterations in the connectivity of functional brain networks [13,14]. 

DM1 is caused by the abnormal expansion of a trinucleotide CTG repeat in 3′UTR of the myotonic dystrophy protein kinase (*DMPK*) gene [15]: in contrast to the short repeats carried by unaffected individuals, pathogenic expansions are longer than 50 CTG repeats, reaching >1000 CTG in the most severe forms of the disease: larger repeats are associated with more severe symptoms and earlier onset [16]. Expanded RNAs containing long CUG repeat tracts accumulate in the nucleus of DM1 cells, forming ribonuclear aggregates (or RNA foci) that perturb the activity of RNA-binding proteins [17]. Among those, muscleblind (MBNL) proteins are sequestered by toxic RNA foci, while CELF (CUG-BP and ETR-3-like factors) family members are upregulated. The dysregulation of these RNA-binding proteins perturbs several gene expression steps, including the alternative splicing of a subset of developmentally regulated exons [18].

In the brain of DM1 patients, RNA foci accumulate in cortical neurons, astrocytes, and oligodendrocytes [19,20]. However, the cellular and molecular pathways behind DM1 brain dysfunction remain elusive, and there is a lack of research studies and strategies for the effective treatment of CNS dysfunction. Relevant animal models of DM1 provide powerful tools to investigate disease neurobiology. We previously generated the transgenic DMSXL line, which expresses expanded *DMPK* transcripts in multiple tissues and cell types and displays multisystemic phenotypes [21,22]. In the CNS, DMSXL mice exhibit nuclear RNA foci in neurons and astrocytes of multiple brain regions, including the frontal and temporal cortex, as well as the hippocampus [21]. The previous analysis of DMSXL mice provided evidence of presynaptic dysfunction, illustrated by the upregulation of RAB3A and abnormal hyperphosphorylation of synapsin-1 (SYN1), two abundant vesicle-associated proteins [20]. DMSXL mice have also revealed signs of glial cell pathology, demonstrated by the downregulation of the astrocyte-specific glutamate transporter SLC1A2 (solute carrier family 1 member 2)—traditionally known as GLT1 (glutamate transporter 1) or EAAT2 (excitatory amino acid transporter 2) [23]. Glutamate is the most abundant excitatory neurotransmitter in the mammalian CNS, and it is involved in a wide range of functions, such as cognition and memory [24]. The homeostasis of extracellular glutamate is therefore critical for proper brain function. Glutamate clearance by GLT1 protects neurons from excessive glutamate stimulation, avoids neurotransmitter spillover and subsequent activation of extrasynaptic receptors, and maintains synapse independence [25]. In line with the low GLT1 expression, glutamate uptake is reduced in primary cultures of DMSXL astrocyte, and neurons show signs hyperexcitability in vivo, demonstrated by increased spontaneous firing of Purkinje neurons [23] and higher susceptibility to induced seizures [26]. 

In this study, we investigated the functional impact of the DM1 mutation on the tight regulated mechanisms of neuronal excitation, which control synaptic transmission and plasticity. To this end, we took advantage of DMSXL mice to study the glutamatergic transmission in the hippocampus, a key brain region essential for the regulation of attention, memory, and emotion, which are frequently impaired in DM1 [3,4]. Lower GLT1 expression and glutamate uptake in the DMSXL hippocampus were associated with increased tonic glutamate currents, suggestive of higher ambient glutamate in the immediate vicinity of membrane receptors. DMSXL mice displayed significant defects in short- and long-term synaptic plasticity. Surprisingly, we also found an intriguing increase in GABA tonic currents and extracellular levels, which suggest a contribution of this neurotransmitter in DM1 brain pathology. 

## 2. Results

### 2.1. Glutamate Uptake Ability in the DMSXL Hippocampus

**Figure 1:**
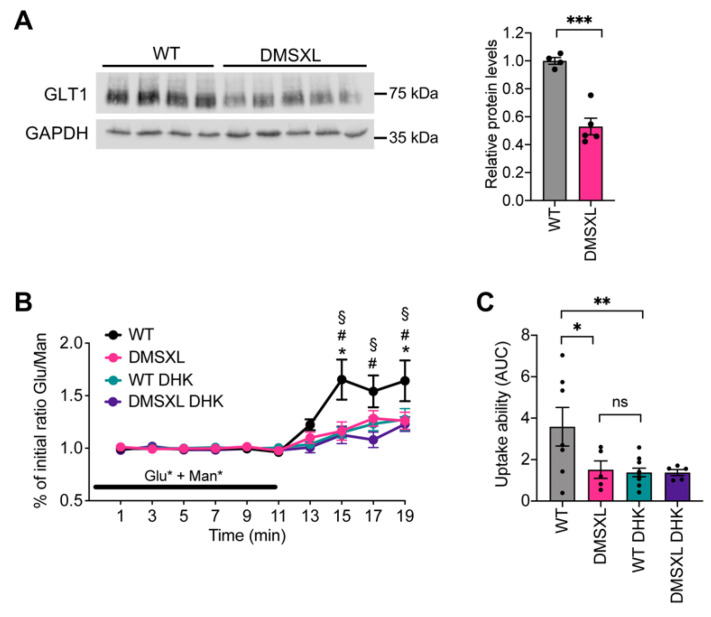
Glutamate uptake in mouse hippocampus. (**A**) Western blot quantification of GLT1 protein levels in the hippocampus of DMSXL and WT mice at 1 month of age. GAPDH was used as loading control. *** *p* < 0.001. (**B**) The in vivo ability of glutamate uptake in the dorsal hippocampus was monitored in mice aged 2–3 months by comparing the ratio of L-[^3^H]-glutamate/[^14^C]-mannitol radioactivity in the presence (DMSXL, *n* = 5; WT, *n* = 10) or absence (DMSXL, *n* = 5; WT, *n* = 7) of 2 mmol/L DHK. Ratios were normalized to the initial isotopic solution infused through the microdialysis probe. * *p* < 0.01, DMSXL vs. WT; ^#^ *p* < 0.05, WT DHK vs. WT; ^§^ *p* < 0.01, DMSXL DHK vs. WT. Glu, glutamate. Man, mannose. (**C**) Estimation of glutamate uptake ability using AUC during the wash-out. * *p* < 0.05, ** *p* < 0.01.

We first investigated GLT1 protein levels in the whole hippocampus of DMSXL mice and the impact on glutamate uptake and extracellular levels. Western blot quantification of GLT1 steady-state levels revealed a significant decrease in the glial glutamate transporter by 50% (*p* < 0.001) (Figure 1A). Glutamate uptake ability was challenged by administrating exogenous glutamate [27,28], simultaneously to nonmetabolizable mannitol [29] as previously described [30] (Supplementary Figure S1). The ratio between the two radio-labelled molecules increased during the wash-out period, peaking 4–6 min after the end of isotopic infusion (Figure 1B). Lower glutamate/mannose ratios were found in DMSXL animals, when compared with WT controls, suggestive of defective glutamate uptake ability in the hippocampus, as previously shown in the frontal cortex, another brain region very relevant for DM1 neuropsychological manifestations [30]. To confirm that the DMSXL traces were indicative of decreased glutamate uptake in the hippocampus, we administered an uptake blocker (dihydrokainate, DHK) concomitantly with the two radio-labelled molecules and measured the glutamate/mannose ratio during the perfusion and wash-out periods. As expected, the unspecific blockade of the glial transporters by DHK decreased the wash-out glutamate/mannose ratio in WT mice to levels that were undistinguishable from those found in the DMSXL hippocampus (Figure 1B). We used the area under the curve (AUC) during the wash-out period as an estimate of glutamate uptake ability in the different mouse groups [30] and confirmed that glutamate uptake was significantly reduced in the DMSXL hippocampus relative to WT controls (Figure 1C).

### 2.2. Neurotransmitter Extracellular Levels

**Figure 2:**
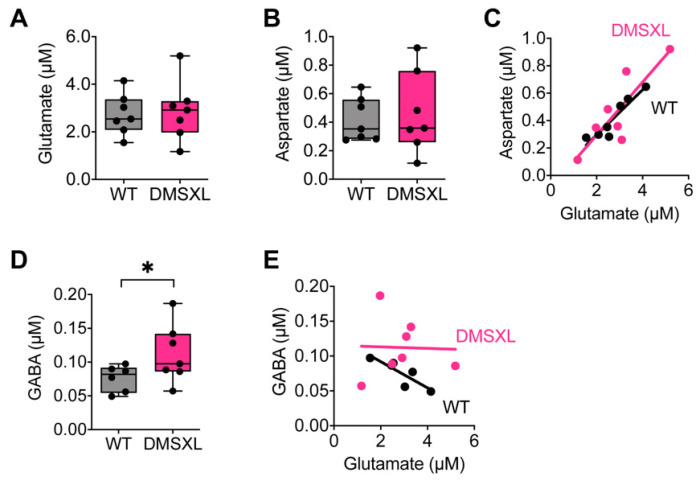
Levels of neurotransmitters in mouse dorsal hippocampus. Extracellular levels of glutamate (**A**) and aspartate (**B**) in DMSXL (*n* = 6) and WT mice (*n* = 7) at 1–2 months of age. (**C**) Correlation between the levels of excitatory glutamate and aspartate in DMSXL mice (R^2^ = 0.73; * *p* = 0.015) and WT controls (R^2^ = 0.88, ** *p* = 0.0019). (**D**) Extracellular levels of GABA in DMSXL and WT mice. * *p* = 0.040. (**E**) Correlation between the levels of inhibitory GABA and excitatory glutamate in DMSXL (R^2^ = 0.001; *p* = 0.95) and WT mice (R^2^ = 0.74; * *p* = 0.027).

We then determined the net extracellular concentration of glutamate in DMSXL mice, using quantitative microdialysis in vivo, as recently described [30]. Notwithstanding the reduced glutamate uptake found in the DMSXL hippocampus, the overall extracellular concentration of glutamate remained unaltered, compared with WT controls (Figure 2A). Similarly, we did not find significant changes in the global extracellular levels of aspartate, another excitatory amino acid taken by the glutamate GLT1 uptake system of glial cells (Figure 2B). The extracellular levels of these two excitatory neurotransmitters were positively correlated in the hippocampus of both genotypes: higher levels of glutamate were associated with higher concentrations of aspartate in individual animals (Figure 2C). We also investigated the extracellular concentration of the inhibitory neurotransmitter GABA and found a surprising increase in DMSXL mice relative to WT controls (DMSXL, 0.112 ± 0.016 µM; WT, 0.076 ± 0.008 µM; *p* = 0.040) (Figure 2D). Importantly, while GABA concentration in individual WT mice was negatively correlated with the levels of glutamate, the correlation was lost in the DMSXL hippocampus (Figure 2E), suggesting a disequilibrium between excitatory and inhibitory neurotransmitters. 

### 2.3. Tonically Activated Receptors by Ambient Neurotransmitters

**Figure 3:**
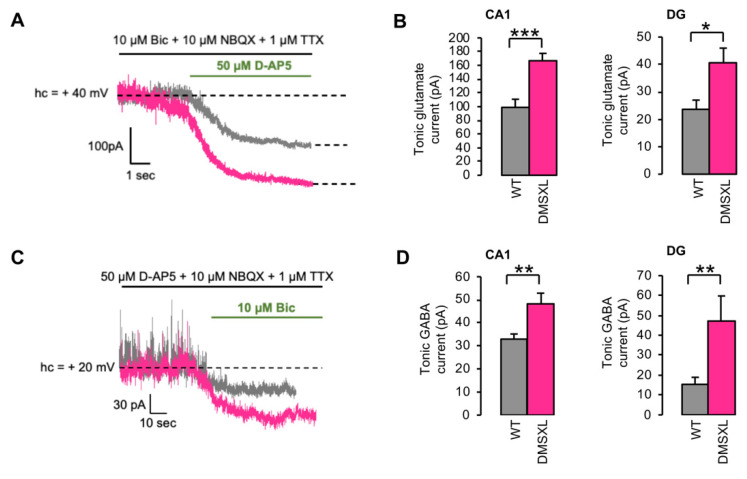
Glutamate and GABA tonic currents in CA1 and DG. (**A**) Representative example of a glutamate NMDA-dependent tonic current, recorded at +40 mV holding potential in CA1, in WT (grey) and DMSXL (magenta) mice at 1–2 months of age. (**B**) The mean amplitude of tonic glutamate currents was evaluated after the action of the NMDAR antagonist D-AP5 and was statistically higher in the CA1 and DG of DMSXL mice (*n* = 8 mice, *n* = 14 neurons), compared with WT controls (*n* = 6 mice, *n* = 13 neurons). CA1, *** *p* = 0.0002; DG, * *p* = 0.012. (**C**) Representative example of a GABA-dependent tonic current, recorded at +20 mV holding potential in DG, in WT (grey) and DMSXL (magenta) mice at 1–2 months of age. (**D**) The mean amplitude of tonic GABAergic currents was evaluated after the action of the GABAA antagonist bicuculline and was significantly higher in both hippocampal areas of DMSXL mice (CA1, *n* = 11 mice, *n* = 17 neurons; DG, *n* = 3 mice, *n* = 7 neurons), relative to WT controls (CA1, *n* = 8 mice, *n* = 17 neurons; DG, *n* = 5 mice, *n* = 13 neurons). CA1, ** *p* = 0.0056; DG, ** *p* = 0.0064.

In vivo microdialysis is capable of quantifying global extracellular glutamate levels, which are the net result from the combination of neurotransmitter release and uptake by neurons and glial cells [27] at the level of a whole tissue. The overall concentration measured by microdialysis (Supplementary Figure S1) may then differ from the ambient extracellular glutamate levels present in the synaptic cleft and extrasynaptically, which can activate membrane receptors. Therefore, we investigated ambient glutamate indirectly by the recording of tonically activated glutamate NMDA receptors (NMDARs) in the hippocampus using whole-cell patch clamp recordings of CA1 (*cornu ammonis 1*) and DG (*dentate gyrus*) neurons. For that purpose, the holding current necessary to maintain the recorded cells at +40 mV was measured in the presence of an inhibitor cocktail (see methods) that spares NMDARs (Figure 3A). At this potential, NMDARs are relieved from their Mg^2+^ block and potentially activable by residual glutamate in the synaptic cleft and away, therefore generating a NMDAR-dependent tonic current [31]. The mean tonic current amplitude measured after the action of the selective NMDAR antagonist D-AP5 was significantly higher in the CA1 area of DMSXL than in WT mice (DMSXL, 166.8 ± 10.7 pA; WT, 98.8 ± 11.8 pA; *p* = 0.0002). In DG, the amplitude of the tonic current was smaller than in CA1 for both genotypes, but still significantly higher in DMSXL when compared with WT mice (DMSXL, 40.8 ± 5.1 pA; WT 23.6 ± 3.3; *p* = 0.012) (Figure 3B). 

Since we found an intriguing elevation of extracellular GABA in the DMSXL hippocampus by in vivo microdialysis in DMSXL mice, we also measured the GABA tonic current in CA1 and DG neurons. The tonic activation of GABA_A_ receptors (illustrated by their blockade by the selective GABA_A_ antagonist bicuculline) was significantly higher in the CA1 area of DMSXL relative to WT mice (DMSXL, 48.2 ± 4.6 pA; WT, 32.9 ± 2.2 pA; *p* = 0.0056). A significant increase in GABA tonic currents was also found in the DG of the DMSXL mice when compared with WT controls (DMSXL, 47.1 ± 12.6 pA; WT, 15.3 ± 3.5 pA; *p* = 0.0064) (Figure 3C). Overall, these results suggest higher extracellular glutamate and GABA levels in CA1 and DG of DMSXL mice. 

We next tested whether the increased glutamate and GABA tonic currents in DMSXL mice could be attributed to an enhanced spontaneous release. To this end, we measured the amplitude and frequency of miniature excitatory (mEPSCs) and inhibitory postsynaptic currents (mIPSCs, respectively, induced by the spontaneous release of glutamate and GABA) in CA1 and DG neurons. The frequency of mIPSCs was significantly higher in DMSXL mice relative to WT controls in CA1 (DMSXL, 10.3± 0.8 Hz; WT, 5.78 ± 0.42 Hz, *p* < 0.0001) and in DG (DMSXL, 3.7 ± 0.7 Hz: WT, 1.54 ± 0.15 Hz, *p* = 0.0015) with no significant change in their amplitude. No differences in the frequency or amplitude of mEPSCs were found between genotypes (Supplementary Figure S2).

### 2.4. Basal Synaptic Transmission and Short-Term Synaptic Plasticity

**Figure 4:**
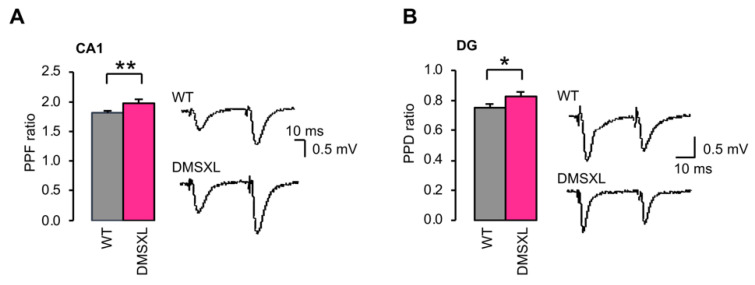
Short-term plasticity in CA1 and DG. (**A**) PPF in CA1. For PPF measurement, the slope of the second response was normalized by the slope of the first one. The PPF ratio was significantly higher in slices from DMSXL mice (*n* = 24 mice, *n* = 55 slices) than in WT controls (*n* = 24 mice, *n* = 58 slices). ** *p* = 0.006. (**B**) PPD in DG. The PPD ratio was significantly increased in DG slices from DMSXL mice (*n* = 7 mice, *n* = 23 slices) relative to WT animals (*n* = 9 mice, *n* = 29 slices). * *p* = 0.038.

We asked whether changes in ambient glutamate and GABA could affect basal neurotransmission in the DMSXL hippocampus. Input/output (I/O) curves of fEPSPs were constructed and revealed no significant difference between WT and DMSXL mice in both regions of the hippocampus studied (Supplementary Figure S3), indicating no change in basal AMPA/kainate receptor-mediated synaptic transmission in response to the CTG repeat expansion. To investigate short-term plasticity in DMSXL mice, we assessed paired-pulse facilitation (PPF) in CA1 and paired-pulse depression (PPD) in DG. PPF and PPD, known to be an index of changes in presynaptic glutamate release, were statistically higher in DMSXL CA1 (DMSXL, 1.97 ± 0.04; WT, 1.81 ± 0.03; *p* = 0.006) and DMSXL DG (DMSXL, 0.83 ± 0.03; WT, 0.75 ± 0.02; *p* = 0.038) (Figure 4). These findings suggest changes in evoked release in both hippocampal structures of transgenic DM1 mice.

### 2.5. Effect of Glutamate Transporter Inhibition on Synaptic Plasticity

**Figure 5:**
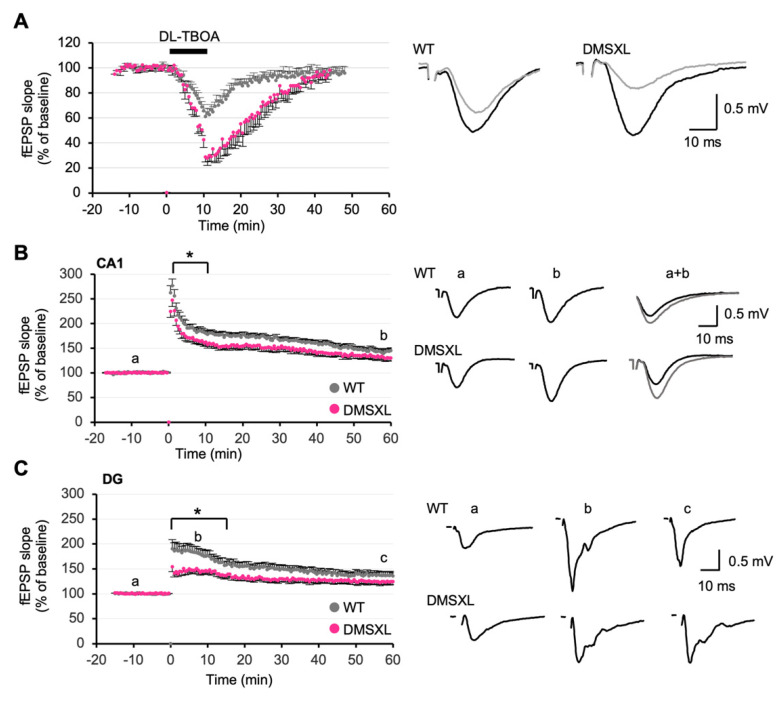
Long-term synaptic plasticity in the DMSXL hippocampus. (**A**) Effects of the inhibitor of glutamate transporter DL-TBOA on fEPSPs. DL-TBOA, applied for 10 min at 40 µM, induced a strong depression of the synaptic transmission in DG of both DMSXL (*n* = 4 mice, *n* = 8 slices) and WT mice (*n* = 5 mice; *n* = 9 slices). The effect was significantly stronger in DMSXL mice, ** *p* = 0.002. Right, sample traces of recordings before (black) and 20 min after TBOA application (grey) are shown. (**B**) LTP in CA1. The time course of LTP following a 3 × 100 Hz stimulation was significantly decreased in hippocampal slices from DMSXL mice (*n* = 9 mice; *n* = 19 slices) as compared with WT controls (*n* = 9 mice; *n* = 19 slices), * *p* = 0.022. The potentiation measured 60 min after stimulation was not different between genotypes, *p* = 0.1. Examples of fEPSP recordings during the baseline (a) and 60 min after induction of LTP (b) are shown on the right. (**C**) LTP in DG. LTP induced by a 2 × 100 Hz stimulus was significantly decreased in DMSXL mice (*n* = 7 mice; *n* = 12 slices) during the early phase postinduction (first 15 min following stimulation) compared with WT (*n* = 6 mice; *n* = 15 slices). *p = 0.019. The mean LTP magnitude during the last 15 min of the recordings was comparable between genotypes, *p* = 0.17. The overall time course of LTP was significantly different in DMSXL mice, *p = 0.047. On the right, individual traces of fEPSPs are shown in the three conditions: before (a), 10 min after stimulation (b), and 60 min after stimulation (c).

Alterations in the homeostasis of extracellular glutamate and GABA can affect the processing of synaptic inputs, with strong repercussions on synaptic plasticity. A depression of synaptic transmission can be induced by the activation of extrasynaptic NMDARs following the inhibition of glutamate transporters [32]. To investigate whether the 50% reduction in GLT1 expression and glutamate uptake found in the DMSXL hippocampus were sufficient to have an impact on this depression, we challenged glutamate uptake by applying the glutamate transporter blocker DL-TBOA to hippocampal slices. A 15 min fEPSP baseline was recorded; then DL-TBOA (40 µM) was bath-applied for 10 min and finally washed out. DL-TBOA induced a reversible depression of the fEPSPs in both WT and DMSXL mice. However, the depression was significantly more pronounced and lasted longer in DMSXL mice (maximum depression: 71.1 ± 3.4%), relative to WT controls (maximum depression: 38.9 ± 4.4%) (*p* = 0.0023) (Figure 5A). In the absence of DL-TBOA, a low-frequency stimulation induced a comparable long-term depression (LTD) in DMSXL mice and WT mice: the mean values registered during the last 15 min of the recordings were not statistically different between DMSXL (69.8 ± 3.6%) and WT mice (66.4 ± 3.2%) (Supplementary Figure S4). These results show DMSXL abnormalities in chemically evoked LTD (triggered by the treatment with DL-TBOA), whereas electrically induced LTD (triggered by low-frequency stimulation) remained unaltered.

### 2.6. Long-Term Synaptic Potentiation

We next evaluated long-term potentiation (LTP). In CA1, high-frequency stimulation induced a mean LTP of 147.5 ± 5.5% of baseline in WT mice (measured between 50 and 60 min after stimulation). In DMSXL mice, the mean value was 134.8 ± 6.3%. This LTP was not significantly different from WT controls (147.5 ± 5.5%) (*p* = 0.1) (Figure 5B). However, when analyzing the whole-time course of LTP, a significant overall decrease was found in DMSXL mice (*p* = 0.022). There was a significant reduction in post-tetanus potentiation strength during the first 15 min after stimulation (*p* = 0.043) (Figure 5B). Interestingly, the analysis of DG also revealed a pronounced effect in the induction of LTP in DMSXL mice. Although the mean LTP magnitude during the last 15 min of the recordings was comparable between DMSXL (125.5 ± 6.2%) and WT mice (137.8 ± 6.9%), the entire time course of LTP was significantly different between both groups (*p* = 0.047) (Figure 5C). Like CA1, the post-tetanic potentiation decay was markedly reduced over the first 15 min that followed high-frequency stimulation (*p* = 0.019) (Figure 5C). 

### 2.7. Effects of NMDA and GABA Antagonists on LTP

**Figure 6:**
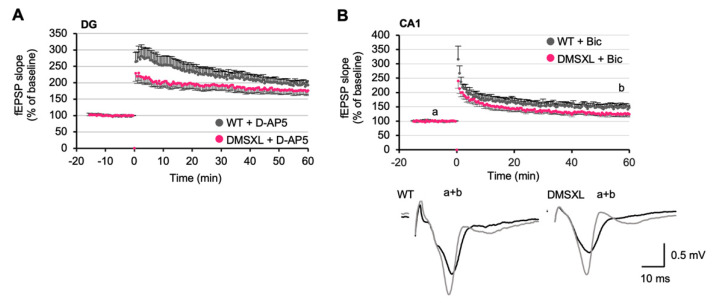
Contribution of NMDA and GABA receptors to altered LTP in DMSXL mice. (**A**) LTP in DG in the presence of the NMDA antagonist D-AP5 (0.5 µM) (DMSXL, *n* = 8 mice, *n* = 18 slices; WT, *n* = 7 mice, *n*= 16 slices). APV was added to the superfusion bath 20 min before high-frequency stimulation and during the entire recording. D-AP5 did not restore the lower strength of LTP in DMSXL mice. (**B**) LTP in the CA1 area in the presence of the GABA_A_ antagonist Bic (4 µM) in DMSXL (*n* = 4 mice, *n* = 9 slices) and WT (*n* = 3 mice, *n* = 7 slices) mice. Bic was added to the perfusion bath 20 min before high-frequency stimulation and during all the recording. Bic did not restore the lower strength of LTP in DMSXL mice. Representative superimposed sample traces of evoked AMPAR-mediated fEPSPs before (a) and after (b) completion of LTP in a slice from WT and a slice from DMSXL mice.

Appropriate glutamate concentration in the synaptic cleft is required for hippocampal LTP induction. We therefore tested whether the LTP impairment in DG of DMSXL mice could be attributed to local glutamate elevation and tonic NMDAR activation. To this end, we applied a low dose of the selective NMDAR antagonist D-AP5, as previously described [33]. Under these conditions, 1 µM D-AP5 did not restore the abnormal LTP of DMSXL mice to WT levels, and the time course of LTP remained statistically different between animal groups (*p* = 0.039) (Figure 6A). Together with the lack of changes in the NMDA-dependent fEPSP/PFV ratio recorded in DMSXL mice (Supplementary Figure S5), these data indicate that the altered synaptic potentiation of DMSXL mice may not be attributed to an inappropriate NMDA receptor activity. Since an elevation of extracellular GABA concentration was found in the DMSXL hippocampus, we performed additional studies in the presence of bicuculline (4 µM Bic) to investigate whether this GABA_A_ receptor antagonist could restore LTP in DMSXL mice. In CA1, the LTP measured over the last 10 min was still slightly lower in DMSXL mice (126.0 ± 8.5%) than in WT controls (148.2 ± 11.1%), but the difference was not statistically significant. However, the overall time course of LTP was statistically different between mouse genotypes (*p* < 0.05) (Figure 6B). The difference in LTP time course between DMSXL and WT mice under these conditions was comparable to the difference found in the absence of Bic (Figure 5B). Therefore, Bic did not reinstate a normal time course of LTP in the hippocampus of DMSXL mice, arguing against a contribution of elevated GABA level in LTP changes.

### 2.8. Regional Distribution of RNA Toxicity in the Hippocampus of DMSXL Mice

**Figure 7:**
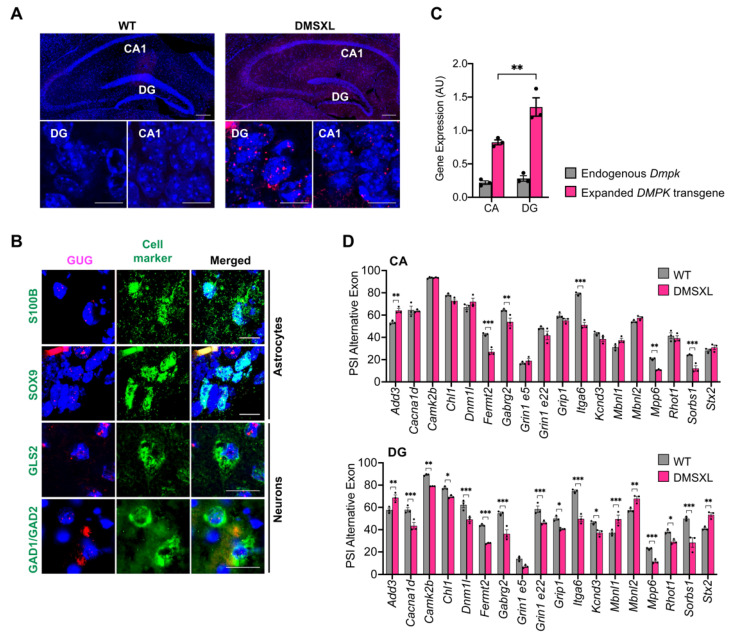
RNA foci accumulation and spliceopathy in the DMSXL hippocampus. (**A**) FISH analysis of nuclear foci accumulation in the hippocampus of WT and DMSXL mice. The DMSXL hippocampus shows greater foci content in DG compared with CA1 at 1 month of age. Scale bar a lower magnification, 200 µm. Scale bar a higher magnification, 50 µM. (**B**) FISH of RNA foci (magenta) combined with IF detection of astrocyte (S100B or SOX9), glutamatergic (GLS2), and GABAergic (GAD1/GAD2) protein markers (green). Nuclei were stained with DAPI (blue). Scale bar, 10 µm. (**C**) Expression of human *DMPK* transgene and mouse endogenous *Dmpk* gene relative to *Polr2a* internal control in CA and DG areas of the DMSXL hippocampus at 1 month of age. ** *p* = 0.0022. (**D**) Splicing dysregulation in the CA and DG areas of the DMSXL hippocampus at 1 month of age (*n* = 3 mice per genotype). The graphs represent the PSI (percentage of splicing inclusion) of alternative exons. * *p* < 0.05, ** *p* < 0.001, *** *p* < 0.001 (two-way ANOVA, Sidak post hoc test for multiple comparisons).

Finally, to gain insight into the molecular mechanism contributing to the synaptic dysfunction found in the DMSXL hippocampus, we examined the accumulation of CUG-containing transcripts. We found toxic ribonuclear inclusions in multiple cells throughout the hippocampus. Interestingly, RNA foci were not homogenously distributed: they were more abundant and seemingly larger in the DG area, relative to CA1 (Figure 7A). Importantly, foci accumulated in glial and neuronal cells of the hippocampus. Among neuronal cells, they were detected in both glutamatergic (expressing the enzyme glutaminase, GLS) and GABAergic neurons (expressing the enzyme glutamate decarboxylase GAD1/GAD2) (Figure 7B). To elucidate the more prominent LTP abnormalities found in DMSXL DG, we performed molecular analysis in DG and CA dissected from a mouse hippocampus (Supplementary Figure S6A). Quantitative real-time PCR revealed that the greater foci content of DMSXL DG was associated with higher expression levels of the human *DMPK* expanded transgene in this region, relative to CA (DMSXL, 1.35 ± 0.14; WT, 0.82 ± 0.04; *p* = 0.0022). In contrast, the expression of the murine *Dmpk* gene did not differ between the two hippocampal regions studied (DG, 0.228 ± 0.04; CA, 0.22 ± 0.03) (Figure 7C). In agreement with the higher expression and accumulation of toxic RNA, DMSXL DG exhibited more pronounced splicing defects of candidate transcripts, with relevant functions in the control of neurotransmission and synaptic plasticity: from the 18 alternative exons selected for this study, 17 were significantly dysregulated in DG, while only 6 were perturbed in CA (Figure 7D and Supplementary Figure S6B). The magnitude of the changes in the splicing of alternative exons was significantly higher in DG relative to CA (*p* < 0.001) (Supplementary Figure S6C). In summary, the DMSXL hippocampus shows pronounced RNA toxicity, notably in the DG regions, characterized by RNA foci accumulation and the dysregulation of relevant genes for synaptic function.

## 3. Discussion

How DM1 affects neurotransmission and synaptic function, leading to the cognitive impairment typical of DM1, has not been fully elucidated. In the present study, we describe for the first time the functional alterations of neurotransmitter homeostasis, related synaptic transmission, and plasticity in the central nervous system of DMSXL mice, a transgenic mouse model of DM1 expressing toxic CUG RNA in the brain. 

### 3.1. Glutamate Uptake and Tonic Currents in DMSXL Hippocampus

DMSXL mice exhibit altered spatial and/or temporal distribution of glutamate and GABA neurotransmitters in the hippocampus, a brain region with abundant RNA foci. Using microdialysis, we showed that glutamate uptake was significantly reduced in the whole DMSXL hippocampus, relative to WT controls. However, this sampling technique failed to detect higher levels of extracellular glutamate in the hippocampus, unlike the frontal cortex of DMSXL mice, where we previously reported increased levels of extracellular glutamate in association with GLT1 downregulation [30]. Despite the unchanged global glutamate levels found in the DMSXL hippocampus by microdialysis, it is conceivable that the spatial resolution of this sampling technique is not sufficient to target specific subregions of the hippocampus (DG vs. CA1). In addition, individual synaptic compartments or membrane microdomains in this complex and heterogeneous brain region cannot be sampled by this approach [34]. To overcome this limitation, we used ex vivo patch clamp techniques on hippocampal slices to specifically investigate events on the membrane of CA1 and DG neurons. We found an increase in tonically activated NMDARs in CA1 and DG, likely due to an elevation of synaptic and extrasynaptic glutamate in both hippocampal areas of DMSXL mice. Interestingly, the increase in glutamate tonic currents detected by patch clamp in the DMSXL hippocampus is similar to the findings previously reported in aged rats [35]. Increased glutamate tonic currents could not be accounted for by higher spontaneous release of this neurotransmitter, which remained unchanged in both hippocampal structures, suggesting that the mechanisms of synaptic glutamate spontaneous release are relatively unaffected in DMSXL mice. It is estimated that 95% of extracellular glutamate reuptake relies on the glial glutamate transporters GLT1/EAAT2 [35]. Hence, our data suggest that the DMSXL increase in tonic current can be attributed to an elevation of extrasynaptic glutamate levels, which results from the ~50% downregulation of the astrocyte-specific GLT1 glutamate transporter and reduced glutamate uptake. It appears, however, that the alterations in glutamate levels are spatially confined to the extrasynaptic space and insufficient to have an impact on the global extracellular glutamate content measured by microdialysis. Importantly, higher ambient glutamate levels are in line with the neuronal hyperexcitability previously reported in DMSXL mice [23] and the increased susceptibility of this line to seizures induced by GABA antagonists [26]. 

Interestingly, in vivo microdialysis revealed an unexpected elevation of extracellular GABA in the hippocampus of DMSXL mice, which was further corroborated by increased tonic GABA currents in CA1 and DG. In contrast to glutamate, higher GABA tonic currents were accompanied by a higher frequency of spontaneous GABA release. In the CNS, the GABAergic system acts in close synergy with the glutamatergic system to control the excitation/inhibition balance. We could imagine that the increase in GABA in DMSXL mice represents an attempt to compensate for glutamate elevation resulting from the downregulation of GTL1 and lower glutamate uptake. However, in contrast with WT mice, extracellular levels of GABA and glutamate did not correlate in DMSXL mice, suggesting a selective presynaptic impact of the DM1 mutation on GABAergic interneurons and a perturbation of the fine equilibrium between neuronal excitation and inhibition, key for proper brain function. Interestingly, we previously reported enhanced basal neurosecretion in cell culture models of DM1, in the absence of stimulation, in association with the abnormal expression and phosphorylation of key synaptic proteins [20]. It is still unclear whether these molecular abnormalities contribute to changes in nonevoked GABA release and how they would specifically affect this neurotransmitter. Although we detected RNA foci accumulation in GABAergic and glutamatergic neurons, our results do not allow us to conclude on the susceptibility of distinct neuronal cell types to expanded and toxic *DMPK* transcripts. A more detailed study is required to address this question and shed light on the neuropathology of DM1 brain disease.

### 3.2. Neurotransmission and Synaptic Plasticity in DMSXL Mice

Electrophysiological analysis of synaptic plasticity in the CA1 and DG hippocampal areas of DMSXL mice was performed to gain insight into the mechanisms of DM1 brain disease. We first demonstrated that PPF and PPD ratios were significantly higher in CA1 and DG, respectively. The paired-pulse ratio is considered a form of short-term plasticity sustained by different mechanisms [36]. Facilitation is prominent at synapses with a low probability of release, meaning that the first stimulus of the pair will not lead to a complete emptying of the readily releasable pool (RRP) of neurotransmitters. When a second stimulus follows shortly (between 20 and 50 ms) after the first, an additional release allows for the increase in the second response due to residual calcium in the presynaptic bouton. The PPF ratio of fEPSPs measured in DMSXL mice was increased, indicating a decrease in the release probability of excitatory glutamate during the first fEPSP. This may reflect changes in the mechanisms underlying PPF, such as a modification of quantal content, or a change in residual presynaptic calcium. We can also hypothesize alterations in the activity of calcium sensors, which promote exocytosis and regulate the mechanisms of facilitation [36]. In DG, the same paired-pulse protocol induces PPD. Depression is observed when the probability of release is high, as is it the case in DG, during the first stimulus of the pair, leading to less available glutamate during the second stimulus. The PPD ratio was increased in DMSXL mice, also suggesting a decrease in the probability of evoked glutamate release.

The glutamate transporter inhibitor TBOA induced a depression of fEPSPs in both mice genotypes, probably due to extrasynaptic NMDAR activation and glutamate spillage, but this chemical depression was stronger in DMSXL mice, in agreement with the altered glutamate clearance. In addition, the time course of LTP was significantly perturbed in DMSXL CA1 and DG, particularly in the short time window that followed tetanic stimulation, suggesting that synapses are less prone to short-term plasticity. As induction of LTP is dependent on optimal synaptic NMDAR activation, we studied pharmacologically isolated NMDA-dependent fEPSPs. No alteration of these responses was observed, suggesting that LTP deficits were not due to a modification in NMDAR activation, as suggested, for instance, in the aging hippocampus [37,38]. A shift in the LTP/LTD threshold in favor of LTD, often associated with a loss of synapses, could also explain the reduced LTP [39]. However, we found no evidence for an increase in electrically induced LTD in DMSXL mice. The electrophysiological phenotypes of young DMSXL mice studied here differ from the milder defects reported in older animals at 4–7 months of age [20], likely due to the reduction of transgene expression with age [40] and the high mortality of the most severely affected animals during the first month [21].

LTP induction and maintenance require optimal extracellular glutamate concentration, which is secured by glutamate transporters, such as GLT1 [41]. Homozygous GLT1 knockout mice exhibit impaired LTP, which is overcome by low concentrations of an NMDAR antagonist [33]. Given the GLT1 downregulation in DMSXL mice, we also tested low doses of D-AP5 on the LTP. However, light NMDAR blockade did not change the time course of LTP in DMSXL animals, suggesting that synaptic NMDARs were relatively spared in these mice, and that the elevation of ambient glutamate was not sufficient to activate extrasynaptic NMDARs. Finally, it has been shown that abnormal spine morphology is associated with a decrease in LTP in mouse models of Alzheimer’s disease (APPxPS1-KI mice) [42] and schizophrenia (Schnurri-2 knockout mice) [43]. Incidentally, altered neuronal morphogenesis was reported in mouse and cell models of DM1 [44,45,46], together with prevalent defects in transcripts and proteins associated with cytoskeleton and cell morphogenesis [47,48,49]. Hence, a detailed morphological analysis of dendritic spines should help elucidate the contribution of ultrastructural neuronal abnormalities to synaptic plasticity and presynaptic function (such as PPF) in DMSXL mice.

### 3.3. RNA Spliceopathy in DMSXL Mouse Brains and Possible Effect on Altered Synaptic Dysfunction

A sustained reduction in LTP was previously reported in a conditional DM1 mouse model expressing high levels of interrupted CUG RNA repeats in the brain [50], and in *Mbnl2* knockouts, in association with altered NMDAR activity and modest mis-splicing of exon 5 of *Grin1* (glutamate ionotropic receptor NMDA-type subunit, also named *Nmdar1*) [26], which regulates receptor potentiation [51]. The higher expression of the interrupted transgene in the conditional mice and the total absence of the MBNL2 protein in the knockout line may explain the more pronounced phenotypes of these DM1 models when compared with our mice. DMSXL mice exhibit normal splicing of the regulatory *Grin1/Nmdar1* exon 5 in CA and DG, but reduced inclusion of exon 22 in DG. Although *Grin1/Nmdar1* exon 22 appears to participate in protein localization to the membrane and in the interaction with other regulatory protein partners [51], NMDAR activity does not appear to be abnormally increased in DMSXL, in contrast with homozygous *Glt1* knockout mice [33]. The functional consequences of exon 22 mis-splicing requires further investigation.

We suggest that impaired synaptic plasticity in the DMSXL hippocampus is the combined result of multiple mis-splicing events (as well as other molecular defects), which together have a significant impact on neurotransmission. Among the genes mis-spliced in the DMSXL hippocampus, many are involved in the control of synaptic function. *Cacna1d/Cav1.3* (a calcium channel) and *Rhot1* (a mitochondrial GTPase) regulate calcium homeostasis, an essential aspect behind neuronal activity [52,53]. Similarly, *Kcnd3/Kv4.3* regulates neuronal excitability and neurotransmitter release through voltage-dependent outward potassium currents [54]. Presynaptic *Add3* and *Stx2* facilitate the localization of synaptic vesicles at the active zone [55,56]. In the postsynaptic membrane, *Camk2b* and *Grip1* regulate the intracellular trafficking of AMPA receptors [57,58,59], *Sorbs1* participates in the clustering of acetylcholine receptors [60], and *Gabrg2* regulates the GABAergic signaling [61]. *Fermt2* encodes a scaffolding protein that regulates axonal growth, PPF, and LTP [62]. Many of the affected genes (e.g., *Add3*, *Cacna1d*, *Camk2b*, *Fermt2, Gabrg2*, *Grip1*, *Kcnd3*, *Sorbs1*, and *Stx2*) are regulated by MBNL proteins, and they are also misregulated in other DM1 mouse models and human tissues [47,63,64,65,66,67]. These data corroborate the involvement of the mis-spliced events studied in DM1 neuropathogenesis, particularly in DG, where the spliceopathy appears to be more pronounced, possibly contributing to the more prominent synaptic dysfunction in this area of the hippocampus relative to CA1. Importantly, DG is a brain region where adult neurogenesis takes place to add new neurons to hippocampal circuits [68]. Defective DG neurogenesis may contribute to brain disease [69], and its role in DM1 neurodysfunction deserves further attention in future investigations.

### 3.4. Conclusion

In conclusion, using a transgenic mouse model of DM1, we found evidence of defective synaptic plasticity and abnormal glutamate and GABA neurotransmission. Neurochemical and electrophysiological defects were associated with localized splicing dysregulation, which was more pronounced in restricted areas of the hippocampus. RNA mis-splicing affected components of the presynaptic membrane (e.g., *Stx2*), postsynaptic receptors (e.g., *Gabrg2*, *Grin1*), and cytoskeleton and scaffold proteins (e.g., *Fermt2*, *Itga6*, and *Sorbs1*). Taken together, the molecular and functional changes in the synaptic environment may represent multiple factors of vulnerability to neuronal dysfunction, which contribute to cognitive impairment and behavioral changes typical of this debilitating disease. Deciphering the relative contribution of misregulated events to the neuropathophysiology of DM1 will require splicing modulation of alternative exons in DMSXL and/or WT mouse brains. The investigation of the physiological consequences of individual mis-splicing events will help identify priority disease targets for therapeutic intervention.

## 4. Materials and Methods

### 4.1. Transgenic Mice

DMSXL transgenic mice (> 98.9% C57BL/6 background) carry 45 kb of human genomic DNA from a DM1 patient [70]. The DMSXL mice used in this study carried more than 1500 CTG repeats within the *DMPK* transgene [22]. DMSXL transgenic status was assessed as previously reported [48]. Animals of both sexes were studied, aged between 30 and 90 days. Mice were housed with food and water ad libitum at a constant temperature and in a 12 h/12 h light cycle. This project was conducted according to the ARRIVE guidelines (Animal Research: Reporting In Vivo Experiments) with authorization for animal experimentation number 75003 in the animal facility with approval number B91228107 both delivered by Prefecture de Police and the French Veterinary Department.

### 4.2. Reagents

The chemical compounds used in this study included the GABA_A_R antagonist bicuculline methiodide (Bic, Abcam, Cambridge, UK), the glutamate uptake inhibitor D,L-threo-beta-benzyloxyaspartic acid (DL-TBOA, Tocris, Illkirch, France), the NMDAR antagonist D-2-amino-5-phosphonopentanoic acid (D-AP5, 30–50 µM, Abcam, Cambridge, UK), the a-amino-3-hydroxy-5-methyl-4-isoxazolepropionic acid receptor (AMPAR) blocker (NBQX, 10 µM, Abcam, Cambridge, UK), the voltage-gated Na^+^ channel blocker tetrodotoxin (TTX, Abcam, Cambridge, UK), and the glutamate uptake inhibitor dihydrokainate (DHK, Abcam, Cambridge, UK).

### 4.3. Cornu Ammonis (CA) and Dentate Gyrus (DG) Dissection from Mouse Hippocampus

CA and DG were dissected from the mouse hippocampus under a stereomicroscope, as described [71]. The identity of the dissected hippocampal areas was confirmed by semiquantitative RT-PCR of CA- and DG-specific genes, *Tyro3* and *Trpc6*, respectively. The primers used were designed using MacVector and are listed in Supplementary Table S1.

### 4.4. Reverse Transcription and PCR Analysis

Reverse transcription, RT-PCR analysis of alternative splicing, and real-time RT-PCR were performed as previously described [48]. Candidate alternative splicing events were selected from previous transcriptomic studies performed in DM1 mouse models and/or human brain tissue [26,47,48,72]. Transcripts with known functions in the regulation of synaptic plasticity and neurotransmission were selected for RT-PCR splicing analysis. Primer sets for PCR were designed using MacVector and are listed in Supplementary Table S1. 

### 4.5. Fluorescent In Situ Hybridization (FISH) and Immunofluorescence Analysis (IFA)

Ribonuclear inclusions were detected using a 5′-Cy3-labelled (CAG)_5_ PNA probe [21], and IFA combined with FISH was performed as previously described [20]. The following primary antibodies were diluted as specified by the manufacturer: GAD1/GAD2 (Abcam, ab183999, Abcam, Cambridge, UK), GLS2 (GeneTex, GTX81012), SOX9 (R&D Systems, AF3075), S100B (Sigma, S2644). Microscope image acquisition and analysis was performed as previously described [48].

### 4.6. Western Blot Analysis 

Total protein extraction, electrophoresis, and immunoblotting were performed as previously described [23] with the following primary antibodies: GLT1, kindly provided by Jeffrey D. Rothstein (Johns Hopkins University, Baltimore, MD, USA), and GAPDH, GeneTex (GTX627408).

### 4.7. Ex Vivo Mouse Brain Slice Preparation

Mice were deeply anesthetized with isoflurane and decapitated. The brain was rapidly removed from the skull and placed in an oxygenated ice-cold (0–3 °C) artificial cerebrospinal fluid (aCSF) containing the following: 124 mM NaCl, 3.5 mM KCl, 1.5 mM MgSO_4_, 2.5 mM CaCl_2_, 26.2 mM NaHCO_3_, 1.2 mM NaH_2_PO_4_, and 11 mM glucose. Sagittal hippocampal slices (400 µm thick) were cut with a Leica 1200S vibroslicer and allowed to recover for at least 1 h before recording in the aCSF solution at 27 °C. Each slice was individually transferred to a submersion-type recording chamber and continuously superfused with the aCSF medium equilibrated with 95% O_2_ and 5% CO_2_.

### 4.8. Extracellular Recordings

Extracellular recordings were obtained at room temperature from the apical dendritic layer of the *cornu ammonis* 1 (CA1) area using micropipettes filled with aCSF. Presynaptic afferent fiber volleys (PFVs) and field excitatory postsynaptic potentials (fEPSPs) were evoked by electrical stimulation of Schaffer collaterals and commissural fibers located in the *stratum radiatum*. For *dentate gyrus* (DG) experiments, stimulation and recording electrodes were both placed in the middle one-third of the DG molecular layer, 500 µm apart.

### 4.9. Synaptic Transmission

Input/output (I/O) curves of fEPSPs were constructed to assess basal synaptic transmission. The slopes of three averaged PFVs and fEPSPs were measured, and the fEPSP/PFV ratio plotted against stimulus intensity. Paired-pulse facilitation (PPF) of synaptic transmission, an electrophysiological paradigm that investigates the presynaptic release of a transmitter, was induced in the CA1 area by electrical stimulation of Schaffer collaterals/commissural fibers with paired pulses at an interstimulus interval of 40 ms. The PPF was quantified as the ratio of the second fEPSP slope over that of the first response. Paired-pulse depression (PPD) of synaptic transmission was obtained in DG by electrical stimulation of the performant path using the same protocol as for PPF in CA1. 

### 4.10. Synaptic Plasticity

A test stimulus was applied every 10 s in control medium and adjusted to get a fEPSP with a baseline slope of 0.1 V/s to investigate LTP. The averaged slope of three fEPSPs was measured for 15 min before high-frequency stimulation (HFS), 3 × 100 Hz, separated by 20 s in CA1 experiments, and 2 × 100 Hz in the presence of the GABA_A_R antagonist Bic (50 µM) in DG experiments. Testing with a single pulse was then resumed for 60 min, and the level of LTP compared with baseline was measured during the last 15 min of the recording. To assess NMDAR-dependent fEPSPs, I/O curves were constructed from recordings obtained in slices perfused with a low-Mg^2+^ (0.1 mM) aCSF supplemented with the non-NMDAR antagonist NBQX, 10 µM.

### 4.11. Patch Clamp Recordings

Whole-cell patch clamp recordings of CA1 pyramidal neurons were obtained at room temperature with borosilicate patch pipettes (open-tip resistance 5 MΩ) filled with an internal solution containing 140 mM CsCH_4_O_3_S, 6 mM CsCl, 2 mM MgCl_2_, 10 mM HEPES, 1.1 mM EGTA, 5 mM QX-314, and 4 mM ATP (pH 7.3; 290–310 mosm). Voltage clamp recordings were performed with an AxoPatch 1-D amplifier (Molecular Devices, San Jose, CA, USA). Signals were filtered at 2 kHz and acquired at a sample rate of 20 kHz using a National Instruments BNC-2090 digitizer and WinLTP software [73]. Access resistance and capacitance were compensated online. Access resistance typically was 10–20 MΩ and remained relatively stable during experiments. 

The tonic glutamate current was assessed in the presence of the GABA_A_R blocker Bic (10 µM), AMPAR blocker (NBQX, 10 µM), and voltage-gated Na^+^ channel blocker tetrodotoxin (TTX, 1 µM). The NMDAR antagonist D-2-amino-5-phosphonopentanoic acid (D-AP5, 50 µM) was bath-applied to evaluate the NMDA component of the holding current (hc) to maintain the recorded neuron at a +40 mV potential. The amplitude of the tonic current was expressed as the difference in the hc before and after application of D-AP5. The tonic GABA current was assessed in the presence of NBQX (10 µM), TTX (1 µM), and D-AP5 (50 µM). Bic was bath-applied to evaluate the GABA component of the hc to maintain the recorded neurons at a 0 mV potential. The amplitude of the tonic current was expressed as the difference in the hc before and after application of Bic. To assess spontaneous release of glutamate, miniature excitatory postsynaptic currents (mEPSCs) were recorded at a holding potential of −60 mV. TTX (1 µM) and Bic (10 µM) were added to the bath to respectively block action potentials and GABA_A_R-mediated inhibitory postsynaptic currents. To assess spontaneous release of GABA, miniature inhibitory postsynaptic currents (mIPSCs) were recorded at a holding potential of 0 mV. NBQX (10 µM), TTX (1 µM), and D-AP5 were added to the bath to respectively block action potentials and ionotropic glutamate receptor-mediated excitatory postsynaptic currents. Miniature EPSCs and IPSCs were analyzed using Spike2 (CED, Cambridge, UK). Peak events were automatically detected using an amplitude threshold of 1.5 times the peak-to-peak baseline noise. The frequency of mEPSC and mIPSC occurrence was estimated during at least 2 min. 

### 4.12. Microdialysis Sampling

Both wild-type (*n* = 20) and DMSXL mice (*n* = 18) were used in experiments according to the current European Communities Council Directives (EU Directive 2010/63/EU for animal experiments). The microdialysis study was performed as previously described and validated [30]. Mice were anesthetized with a mixture of medetomidine chlorhydrate (1 mg/kg)/ketamine (75 mg/kg), and anesthesia was maintained for 5 h with ketamine: at t + 1h and t + 2h, ½-dose, at t + 3h and t + 4h, ¼ -dose. The body temperature was kept constant using a heating pad. Once deep anesthesia was verified through the absence of hind paw withdrawal after pinching, the mouse was placed in a stereotaxic frame (David Kopf, David Kopf Instruments, Tujunga, CA, USA), the skin was incised, and the skull was cleaned and exposed. A hole was drilled, and the meninges were resected to allow the implantation of a home-made dialysis probe (MWCO 6,000 Da, 1 mm active membrane, 225 mm o.d., infused at 1 µL/min) in the right dorsal hippocampus at the following coordinates related to bregma: AP −2 mm, ML 1 mm, DV −1.9 mm from dura for WT mice [74], those coordinates being adapted to AP −1.9 mm (−1.5 mm for mice < 16 g), ML 0.95 mm, and DV −1.9 mm (−1.75 mm for the smallest mice) from the dura of DMSXL mice. Ninety minutes postimplantation, the flow rate was lowered to 1.5 µL/min to initiate zero-flow experiments at t = 2 h 45 min postimplantation. Briefly, artificial cerebrospinal fluid (aCSF) (149 mmol/L NaCl, 2.80 mmol/L KCl, 1.2 mmol/L MgCl_2_, 1.2 mmol/L CaCl_2_, 2.78 mmol/L phosphate buffer, pH 7.4, made with sterile ultrapure water and 0.22 µm filtered) was perfused at 5 successive flow rates (1.5, 1.2, 1, 0.6, 0.3 µL/min) as follows: 5 min stabilization time (except 10 min for 0.3 µL/min), followed by a sampling time (2, 2.5, 3, 5, 10) allowing for collecting 3 µL samples in triplicate, respectively. Then, the flow rate was reinitiated to 1 µL/min for the second part of the experiment. After the collection of 3 2 min samples, a final solution of 100 μmol/L ^3^H-Glu (49.7 Ci/mmol) and 120 μmol/L ^14^C-mannitol (Man) (57.2 mCi/mmol) (PerkinElmer) was infused in the probe during 10 min while collecting 5 other samples, and normal aCSF perfusion was then restored for the collection of 7 last samples during that wash-out period. Samples collected in sterile PCR tubes were stored at −30 °C until CE-LIFD analysis and at −20 °C until isotopic quantification. At the end of the experiment, 0.1% methylene blue was perfused to tag the membrane tract and verify the probe location (Supplementary Figure S1). The ratio of disintegrations per minute (dpm) between Glu and Man was calculated and normalized relative to the initial ratio of the isotopic solution. An area-under-the-curve (AUC) analysis was also performed after the isotopic challenge with ^3^H-Glu and ^14^C-Man.

### 4.13. Dialysate Content Analysis 

For the zero-flow (ZF) experiments, glutamate, aspartate, and GABA levels were determined using capillary electrophoresis with laser-induced fluorescence detection (CE-LIFD) as previously described [30]: 3 μL of sample and 3 μL of standard solutions were derivatized at room temperature by adding 1.2 μL of a mixture (1:2:1 *v*/*v*/*v*) of (i) internal standard (100 µmol/L cysteic acid in 0.117 mol/L perchloric acid), (ii) borate/NaCN solution (100:20 *v*/*v* mixture of 500 mmol/L borate buffer, pH 8.7, and 87 mmol/L NaCN in water), and (iii) 2.925 mmol/L solution of naphthalene-2,3-dicarboxaldehyde in acetonitrile/water (50:50 *v*/*v*). The samples were then analyzed using an automatic capillary zone electrophoresis P/ACE™ MDQ system (Beckman, Brea, CA, USA) equipped with a Zetalif laser-induced fluorescence detector (Picometrics, Labege, France). Excitation was performed using a diode laser (Melles Griot, Carlsbad, CA, USA) at a wavelength of 410 nm. Separations were carried out on a 63 cm × 50 μm i.d. fused-silica capillary (Composite Metal Services, Worcester, England) with an effective length of 52 cm. The day before the analyses, the capillary was sequentially flushed with 0.25 mol/L NaOH (15 min), ultrapure water (15 min), and running buffer (75 mmol/L sodium borate, pH 9.20 ± 0.02, containing 10 mmol/L HP-β-CD and 70 mmol/L SDS) (5 min). The separation was conducted with an applied voltage of 25 kV, a hydrodynamic sample injection (10 s at 0.6 psi), and a temperature between 30 and 45 °C. The capillary was sequentially flushed with 0.25 mol/L NaOH, ultrapure water, and running buffer between analyses (30 s each). Electropherograms were acquired at 15 Hz using P/ACE™ MDQ software [75]. The cconcentrations of glutamate and GABA in the dialysate samples (C_out_) obtained during perfusion under various flow rates (FR) were used to construct an exponential regression plot of C_out_ = C_ext_ × exp^-K*FR^ with C_ext_ as the true extracellular concentration when the FR is zero and K as a constant value as previously used by other authors [76,77].

### 4.14. Statistical Analysis

Statistical analyses were performed with Prism (GraphPad Software Inc., San Diego, CA, USA) or the (BrainPower Inc., Fremont, CA, USA). Data are presented as mean ± standard error of means (±SEM). After performing a normality test on the numeric variables, we used two-tailed Student’s *t*-test for parametric data and the Mann–Whitney *U* test for nonparametric data when 2 groups were compared. When 3 or more groups were compared, we performed a one-way ANOVA or repeated-measures ANOVA. We used two-way ANOVA to assess the statistical interaction between 2 independent variables. Sidak post hoc pairwise comparisons were performed to account for multiple comparisons. * *p* < 0.05, ** *p* < 0.01, *** *p* < 0.001. For the analysis of LTP and LTD, *p*-values were calculated using multivariate ANOVA, followed by Tukey’s post hoc tests to account for the correlations inherent to repeated measures in electrophysiological recordings.

## Supplementary Materials

The following supporting information can be downloaded at https://www.mdpi.com/article/10.3390/ijms23020592/s1.
