# Supplementary material for: DM1 Transgenic Mice Exhibit Abnormal Neurotransmitter Homeostasis and Synaptic Plasticity in Association with RNA Foci and Mis-Splicing in the Hippocampus
Source: Int J Mol Sci. 2022 Jan 6;23(2):592. doi: 10.3390/ijms23020592 (PMC8775431; doi:10.3390/ijms23020592)
Supplement: Supplementary file 1 [file ijms-23-00592-s001.zip › ijms-1538316-supplementary.pdf]

## Supplementary Figures

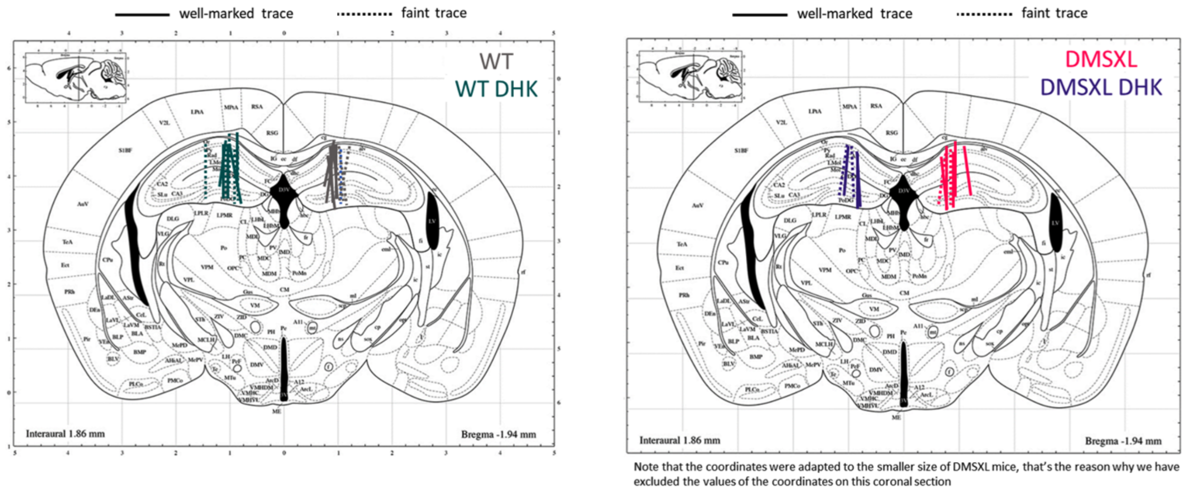


**Supplementary Figure S1. Schematic representation of the traces left in the mouse brain after the removal of the microdialysis probes.**
The probes were previously infused with a 1%-blue methylene solution and the traces were reported on the brain mouse atlas [1]. Note that the probes were all implanted in the right hemisphere. Here, the traces are drawn on both sides of the brain for clarity and direct comparison. Only correct placements are shown. Three DMSXL mice were implanted outside their hippocampus, likely due to their small size and weight (<14 g); two other DMSXL mice and one WT mouse died prematurely while being anaesthesia. In total, 13 DMSXL mice (aged 76 ± 14 days, weight 18 ± 4 g) and 19 WT mice (aged 77 ± 13 days, weight 24 ± 4 g) were included in the microdialysis study.

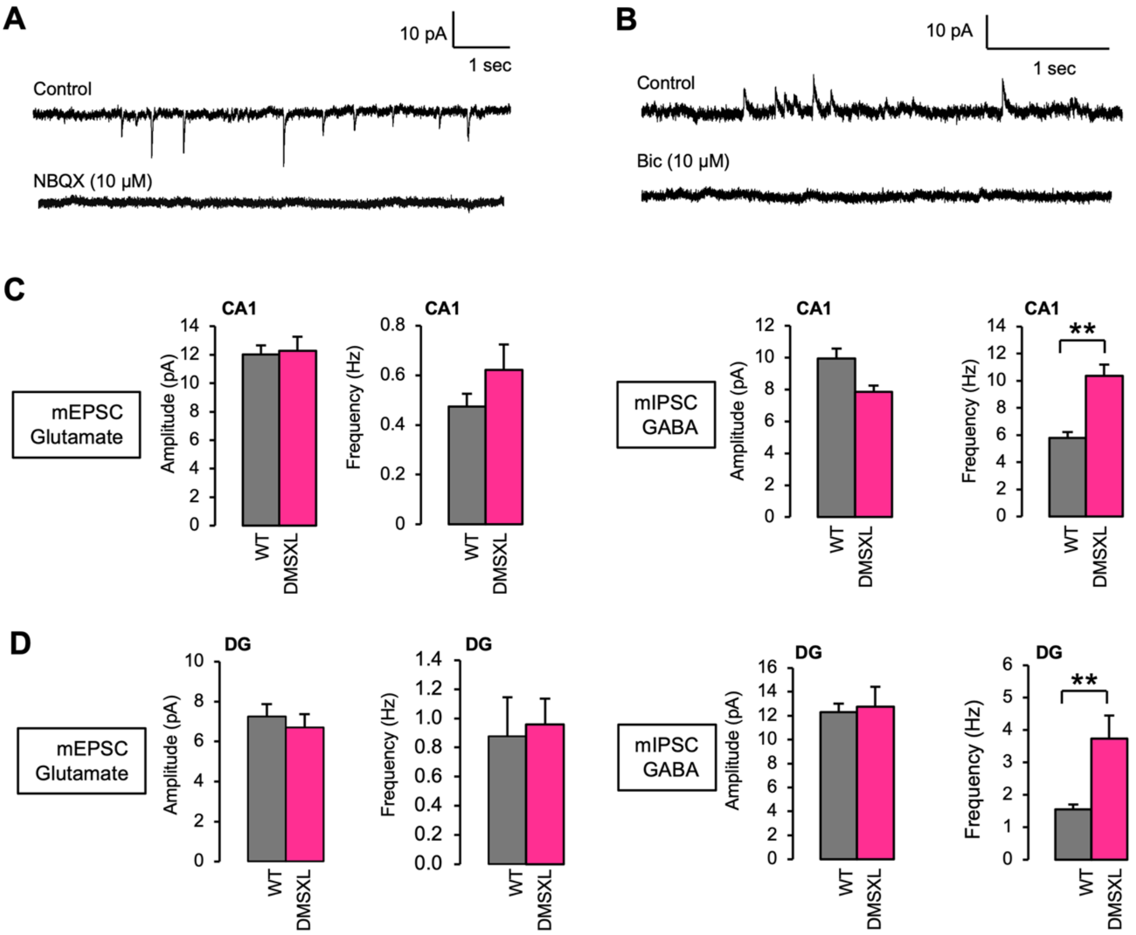


**Supplementary Figure S2. Spontaneous neurotransmitter release: analysis of mini EPSC and mini IPSC**

(**A**) Sample traces of mESCP registered in CA1. Glutamate-dependent mEPSC are blocked by 10 $\mu$M NBQX. (**B**) Sample traces of mIPSC registered in CA1. GABA-dependent mIPSC are blocked by 10 $\mu$M Bicuculline. (**C**) Amplitude and frequency of mEPSC and mIPSC in CA1 of DMSXL and WT mice. (**D**) Amplitude and frequency of mEPSC and mIPSC in DG of DMSXL and WT mice. DMSXL, N = 3-11 mice; n = 8-19 neurons; WT, N = 5-8 mice, n = 11-19 neurons). $^{**}p<0.01$.

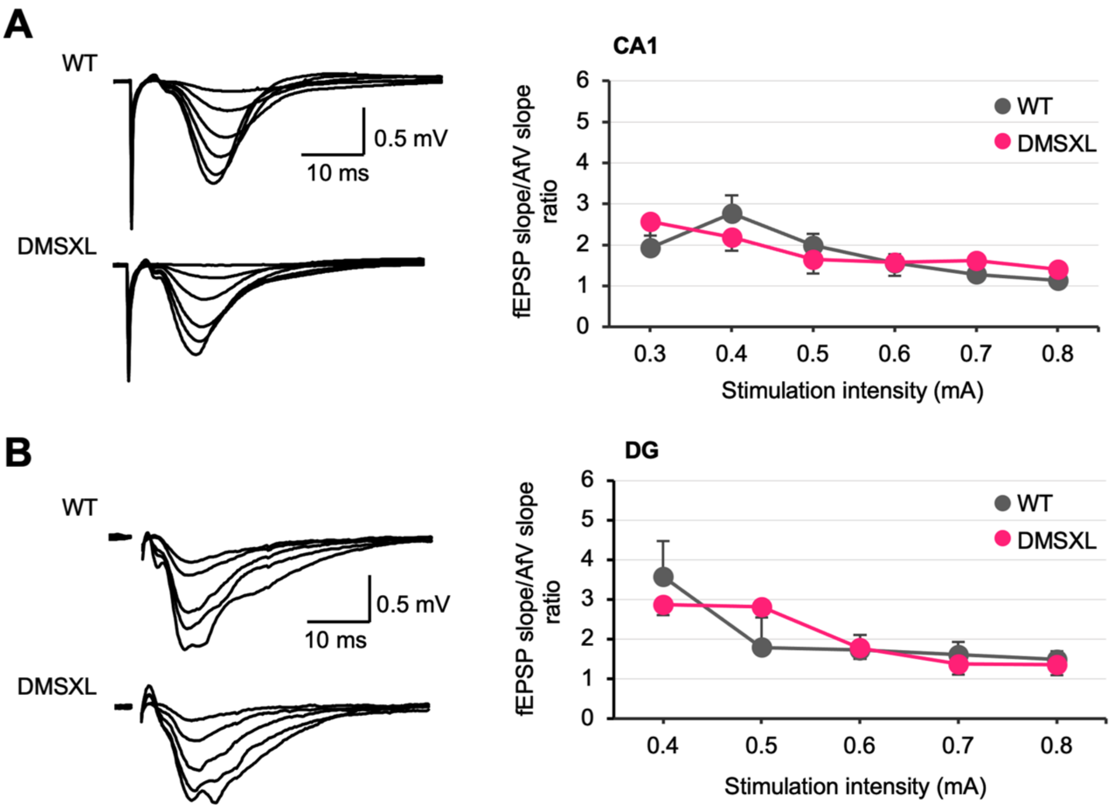


**Supplementary Figure S3. I/O curves in mouse hippocampal slices.**
(**A**) Superimposed sample traces of evoked AMPA receptor (AMPAR) mediated fEPSPs induced by the electrical stimulation of glutamatergic afferents in the *stratum radiatum* of CA1 area. On the right, comparison of the fEPSP slope/PFV slope ratio calculated for stimulus intensities varying from 0.3 to 0.8 mA in WT (N=12 mice, n=20 slices) and DMSXL mice (N=11 mice; n=17 slices). (**B**) Superimposed sample traces of evoked AMPAR-mediated fEPSPs induced by the electrical stimulation of glutamatergic afferents in the DG molecular layer. On the right, comparison of the fEPSP slope/PFV slope ratio measured in DG, calculated for stimulus intensities varying from 0.4 to 0.8 mA in WT (N=8 mice, n=16 slices) and DMSXL mice (N= 5 mice; n=15 slices). No statistically significant differences were found in CA1 ($p$=0.58) and in DG ($p$=0.60).

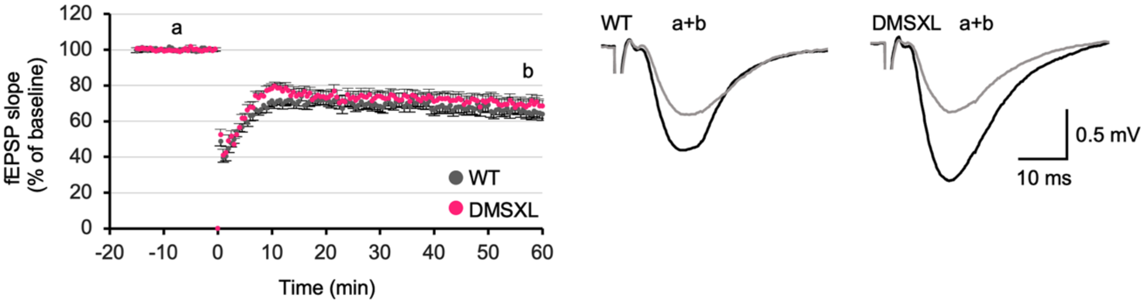


**Supplementary Figure S4. Long-term depression in mouse hippocampus.**
LTD induction in mouse CA1 by low frequency stimulation (2 Hz for 10 min). Recording was stopped during the 10 min conditioning stimulation and resumed after completion of LFS. LFS induced a strong depression of the fEPSP slope, which recovered partially to reach a stable level of depression about 20 min after stimulation. LTD values were measured 50 to 60 minutes after the end of the LFS. We observed a comparable depression in WT and DMSXL mice (WT, N=12 mice, n=18 slices; WT, N=11 mice, n=17 slices), $p$=0.82.

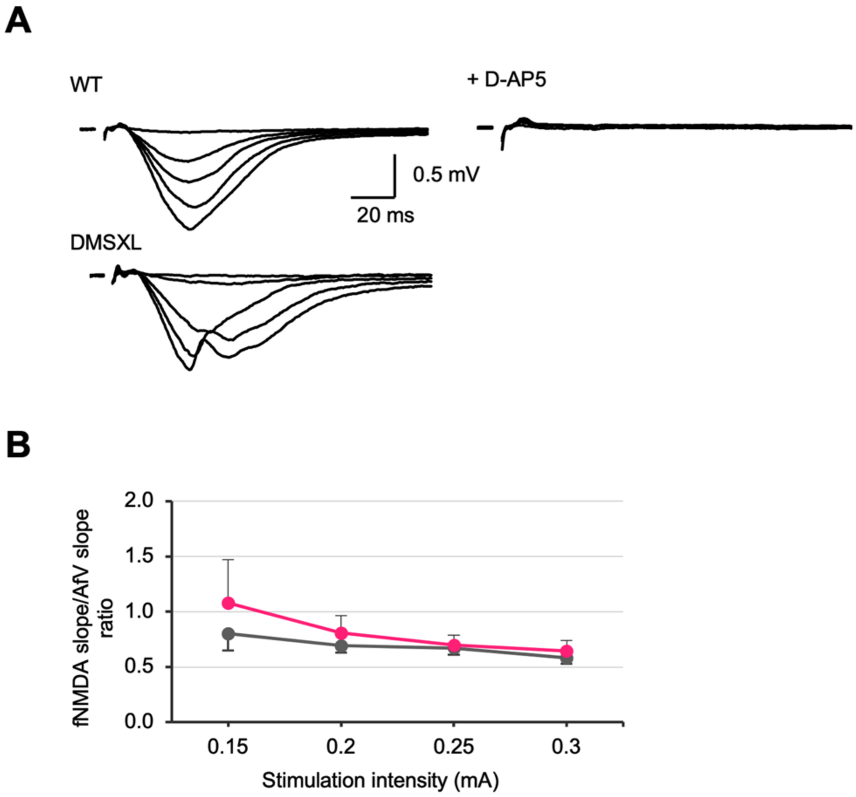


**Supplementary Figure S5. Contribution of NMDAR to altered synaptic plasticity in hippocampus.**

(**A**) Superimposed sample traces of evoked NMDAR-mediated fEPSPs (fNMDA) recorded from WT and DMSXL slices using increasing stimulus intensities. The NMDA nature of this field was demonstrated by its blockade by D-AP5 (50 $\mu$M). (**B**) The comparison of fNMDA/PFV ratio in slices from DMSXL (N=8 mice, n=20 slices) and WT (N=7 mice, n=16 slices) revealed no significant difference between genotypes, *p*=0.58.

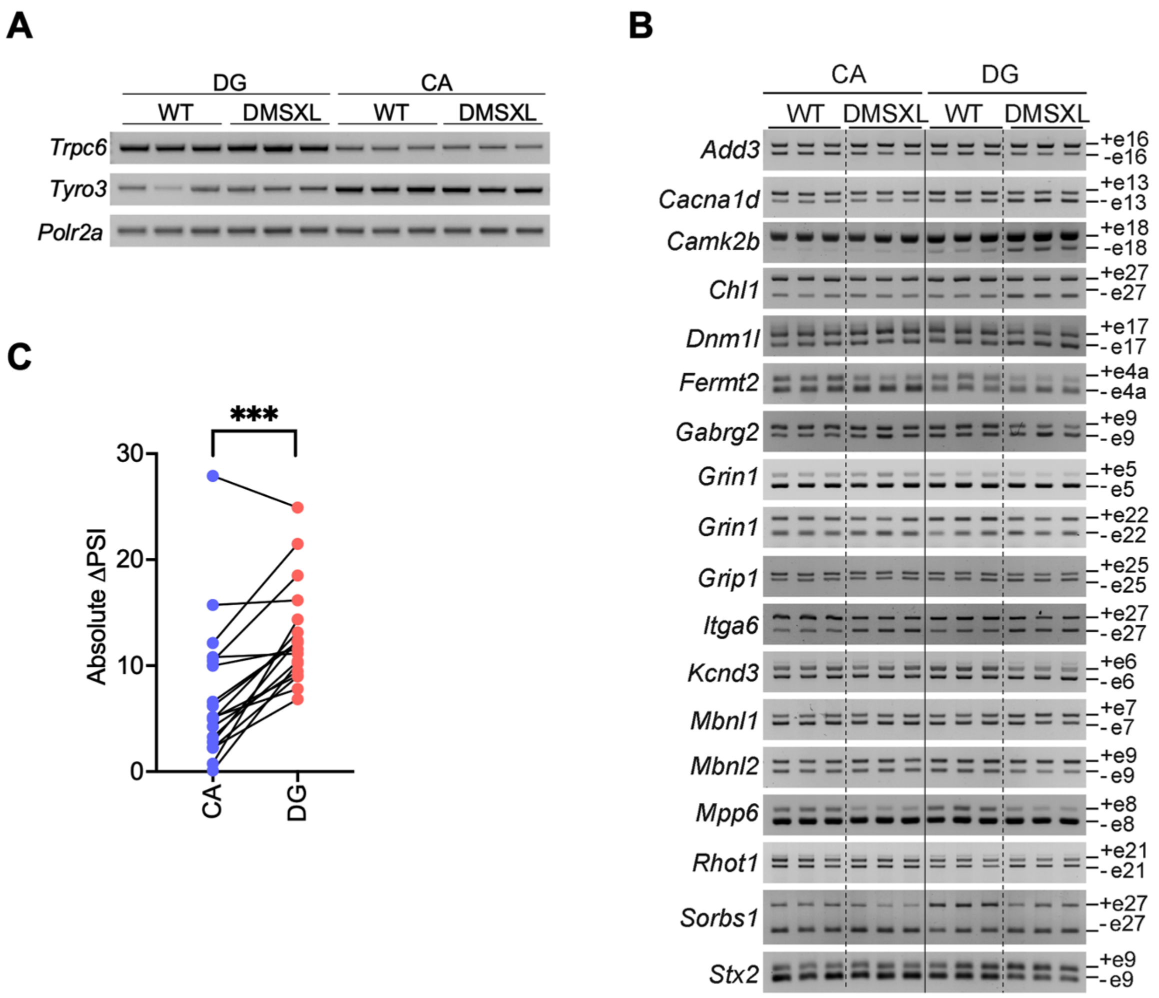


**Supplementary Figure S6. RT-PCR analysis of alternative splicing in DMSXL hippocampus.** (**A**) Semi-quantitative expression analysis of *Trpc6* and *Tyro3* in DG and CA dissected from the brain of 1-month-old mice. The housekeeping gene *Polr2a* was used as loading control. The expression of DG-specific *Trpc6* was higher in DG tissue samples, while the levels of CA-specific *Tyro3* transcripts were increased in dissected CA samples, confirming the enrichment of the dissected tissue samples for these two individual hippocampal regions. (**B**) Representative RT-PCR splicing analysis of candidate transcripts in the CA and DG areas of DMSXL and WT hippocampus at 1 month of age. Alternative exons are indicated on the right. (**C**) Comparison of the magnitude of splicing defects in CA and DG, assessed by the absolute difference in the PSI of selected exons between DMSXL and WT mice. The absolute differences in PSI were significantly higher in DG relative to CA, ****p*<0.001 (Wilcox matched pairs signed rank test).

## Supplementary Tables

**Supplementary Table S1.** Oligonucleotide primer sequences for semi-quantitative RT-PCR analysis.

| Genes | Alternative exon[a] | Size (bp) | Oligonucleotide primer sequences |
|---|---|---|---|
| *Add3* | 16 | 96 | TCCTCCTAACCCATTCAGCCAC<br>GCATCTCGTCCTTGCCGTTC |
| *Cacna1d* | 13 | 60 | CATGCCCACCAGCGAGACTGAA<br>CACCAGGACAATCACCAGCCAGTAAA |
| *Camk2b* | 18 | 45 | CAGGAGACTGTGGAATGTCTG<br>GGCATCTTCATCCTCTATGGTTG |
| *Chl1* | 27 | 162 | TGGCATCTCTCCAACCTCAACTC<br>CCACCTCTGTTCCTCTTCACAAAG |
| *Fermt2* | 4a | 36 | GCTTGAGCTGGAAGGACCTCTTATC<br>GCAGAAGTTGGTGACAAAGGGC |
| *Gabrg2* | 9 | 24 | GTTTTCTGCTTTGGTGGAGTATGG<br>CTTGAAGGTGTGTGGCATTGTTC |
| *Grin1* | 4 | 63 | AGCGTCTGGTTTGAGATGATGC<br>CGTCACATTCTTGGTTCCTGGG |
| *Grin1* | 20 | 111 | CGTGAACGTGTGGAGGAAGAACC<br>TGACCGAGGGATCTGAGAGGTTG |
| *Grip1* | 25 | 45 | TTGTAGGGGCTTCTGACAGTGC<br>GTGTTGCTGCGAGTTGTTTGG |
| *Itga6* | 27 | 130 | GGGATTCTGATGCTGGCTCTATTAG<br>GGCTTTGGGTAGTGTGAGGTGTTC |
| *Kcnd3* | 6 | 57 | CCAGAAGAGGAGCAGATGGGC<br>CAGCAGGTGGTGGTGAGGC |
| *Mbnl1* | 7 | 54 | GCTGCCCAATACCAGGTCAAC<br>TGGTGGGAGAAATGCTGTATGC |
| *Mbnl2* | 9 | 54 | ACCGTAACCGTTTGTATGGATTAC<br>CTTTGGTAAGGGATGAAGAGCAC |
| *Mpp6* | 8 | 42 | TCGACAAGGTCTGCTTCACG<br>TAGTCCTGCTTCTTTGCAGGG |
| *Rhot1* | 21 | 123 | TGCCTCCACCTCAAGCCTTC<br>AATCATCGCTGTTTCAGTAGTGCTC |
| *Sorbs1* | 27 | 168 | CCAGCTGATTACTTGGAGTCCACAGAAG<br>GTTCACCTTCATACCAGTTCTGGTCAATC |
| *Stx2* | 9 | 28 | CTGCACGAGATGTTCATGGATATG<br>TGGCTTTCTTCGTCTCTTCCTTG |
| *Trpc6* | N/A | N/A | GCCTTTATGATTGGAATGTTCAACC<br>GACAGGAAGTGTTCTCCCCTCC |
| *Tyro3* | N/A | N/A | TGGTGGAGAGGACTCACTAAGGTTG<br>CAGGTAAAAGGTGGCACAGGAAC |

[a] Alternative exons are numbered according to the FasterDB web interface (http://fasterdb.ens-lyon.fr/faster/home.pl)

## References


1. Paxinos, G.; B.Franklin, K.; BJ.Frankin, K. *The mouse brain steriotaxic coordinates*; Elsevier Science & Technology Books, 2001; Academic Press, ISBN 9780125476379.
